# Developing the Patient Experience Assessment Questionnaire for Hospital Inpatient Care in South Korea

**DOI:** 10.1111/hex.70560

**Published:** 2026-02-03

**Authors:** Yeongchae Song, Jung‐Eun Kim, Bon Mi Koo, Young‐Geun Choi, Un‐Na Kim, Jin Yong Lee, Min‐Woo Jo, Minsu Ock, Young Kyung Do

**Affiliations:** ^1^ Department of Health Policy and Management Seoul National University College of Medicine Seoul Republic of Korea; ^2^ Review and Assessment Research Department Health Insurance Review and Assessment Service Wonju Gangwon‐do Republic of Korea; ^3^ Korea Ministry of Health and Welfare Sejong Republic of Korea; ^4^ Institute of Health Policy and Management Seoul National University Medical Research Center Seoul Republic of Korea; ^5^ Department of Mathematics Education Sungkyunkwan University Seoul Republic of Korea; ^6^ Department of Preventive Medicine Asan Medical Center, University of Ulsan College of Medicine Seoul Republic of Korea; ^7^ Department of Preventive Medicine Ulsan University Hospital, University of Ulsan College of Medicine Ulsan Republic of Korea

**Keywords:** patient engagement, patient experience, patient reported experience measures, patient‐centred care, quality measurement, questionnaire development

## Abstract

**Background:**

Measuring patient experiences has become a key initiative for improving healthcare quality worldwide. Since patient experiences are inherently shaped by sociocultural context and healthcare system structures, developing locally relevant measurement tools is crucial for reflecting patients' needs and expectations. South Korea has developed a patient experience questionnaire for hospital inpatient care within its National Health Insurance system through a systematic process involving patient engagement. In outlining the development process of the questionnaire, this study assesses its psychometric properties, focusing on validity and reliability.

**Methods:**

The Patient Experience Assessment (PXA) questionnaire was developed through a three‐phase process: (1) item generation via a literature review and qualitative research with patient and caregiver groups, (2) expert review using the Delphi method, and (3) validity and reliability testing through a pilot test. After stakeholder feedback, the current version of PXA includes 21 items across 6 domains. Psychometric properties were assessed using data from 629 inpatients in four general hospitals, split into two subsamples for exploratory factor analysis (EFA) and confirmatory factor analysis (CFA) to ensure robust construct validation. Internal consistency was assessed using Cronbach's alpha.

**Results:**

The PXA questionnaire includes globally recognised patient‐centred themes while addressing unique issues that matter to patients in the Korean healthcare system. EFA identified a four‐factor structure: (1) Information, dignity, and autonomy; (2) Communication with nurses; (3) Communication with doctors; and (4) Hospital environment. CFA confirmed this structure, demonstrating high convergent validity (standardised loadings > 0.70) and satisfactory model fit (CFI and TLI > 0.950, RMSEA = 0.036, SRMR = 0.046). The instrument showed excellent reliability (Cronbach's alpha: total = 0.95; subscales = 0.89–0.93).

**Conclusion:**

The PXA questionnaire, currently being implemented in South Korea, demonstrates robust psychometric properties. This work also exemplifies the process of developing a locally relevant patient experience questionnaire, grounded in global knowledge on patient‐centred care.

**Patient or Public Contribution:**

Patients and caregivers participated in focus group discussions during PXA development. Their opinions directly informed the identification of culturally specific patient experience dimensions relevant to the Korean healthcare system. These findings emphasise engaging target patient populations in developing locally relevant patient experience instruments.

**Abbreviations**
AVE: average variance extracted
CFA: confirmatory factor analysis
CFI: Comparative Fit Index
EFA: exploratory factor analysis
HIRA: Health Insurance Review and Assessment Service
KMO: Kaiser‐Meyer‐Olkin
PXA: patient experience assessment
RMSEA: root mean square error of approximation
SRMR: standardised root mean square residual
TLI: Tucker‐Lewis Index
VOC: voice of the customer

## 1 Introduction

Patient experience has become a key indicator for evaluating healthcare quality, offering a more comprehensive assessment of system performance through the patient's perspectives [1, 2, 3, 4]. To incorporate patient voices into quality assessment and improvement initiatives, many healthcare systems have implemented systematic approaches to measuring and reporting patient experiences [3, 5, 6, 7, 8, 9, 10]. Reflecting this global trend, South Korea has also increasingly recognised the importance of integrating patient perspectives into healthcare quality assessment in recent years [11, 12, 13].

Adapting established international instruments through translation is a widely adopted strategy for selecting patient experience measurement tools [14, 15]. This approach offers several advantages, including validated psychometric properties, cost‐effectiveness, and the ability to facilitate cross‐national comparisons. Similar efforts have been made in South Korea [13]. However, this method may not fully capture the nuances of local healthcare cultures and system‐specific characteristics, as patient experience, as well as their needs and expectations, are inherently shaped by sociocultural context and healthcare system structures [3, 16, 17]. A previous study has empirically demonstrated these limitations, reporting high non‐response rates for items that lacked cultural relevance or were misaligned with local healthcare realities [13].

In response to these limitations, South Korea developed a culturally relevant patient experience measurement tool through a systematic, stakeholder‐engaged process. In 2014, the Health Insurance Review and Assessment Service (HIRA), the national healthcare quality agency, initiated this development project [11]. The methodology encompassed a comprehensive literature review and focus group discussions with patients and caregivers to identify key components and generate initial survey items [12]. Expert consensus was achieved using the Delphi method, and the psychometric properties of the tool were validated through pilot testing. As a result, the Patient Experience Assessment (PXA) questionnaire for hospital inpatient care was established, comprising 6 domains and 21 items [18]. Nationwide implementation of the PXA began in 2017 with the first patient experience survey. This biennial survey reached its fourth iteration in 2023, targeting all general hospitals. Survey results are disseminated to participating institutions and made publicly available through HIRA's official portal to support quality improvement efforts. In addition, the data enable in‐depth analysis at both the individual hospital and healthcare system levels, providing valuable insights for developing improvement strategies [18].

While the tool's psychometric properties were validated during its development [11], several contextual changes have emerged since its nationwide implementation. First, several items and response scales were revised based on stakeholder feedback to improve survey feasibility. Second, changes in the healthcare environment and sociocultural shifts in South Korea may have influenced patient experience and perceptions. These contextual changes, combined with stakeholder demands for updated validation, prompted comprehensive reassessment [19].

This study focuses on reassessing the psychometric properties of the PXA questionnaire to ensure its continued validity in measuring patient experience within the evolving healthcare context. Specifically, the aims are as follows: (1) evaluate construct validity by reassessing the factor structure through exploratory and confirmatory factor analyses, as well as examining convergent and discriminant validity; and (2) assess reliability through internal consistency measures. By comprehensively documenting the development and reassessment process, this study aims to ensure measurement validity within national patient experience programmes and to provide valuable methodological insights for other countries developing culturally relevant patient experience measurement tools.

## 2 Methods

To contextualise the current reassessment, we first outline the trajectory of the PXA questionnaire's development and implementation. The development comprised three distinct phases: (1) Development of the initial PXA questionnaire (2014–2015); (2) Practical refinement during nationwide survey implementation (2016–2022), which involved operational adjustments rather than methodological revisions; and (3) Reassessment of psychometric properties (2022)—the focus of this study (see Appendix for a complete developmental timeline). The first and third phases represent methodologically rigorous scholarly endeavours, while the second phase reflects pragmatic adaptations for large‐scale deployment.

### 2.1 Development of the Initial PXA Questionnaire (2014–2015)

The Patient Experience Assessment (PXA) questionnaire for hospital inpatient care was initially developed in 2014 through a systematic three‐phase process as follows.

#### 2.1.1 Phase 1: Item Generation (2014)

We conducted a comprehensive literature review of previously published conceptual frameworks of patient‐centredness and patient experience measures to identify key domains and items. To ensure cultural relevance, we also collected qualitative data through multiple sources: (a) interviews with six healthcare professionals with expertise in patient experience research or roles in hospital customer satisfaction departments, (b) two focus group discussions, each consisting of six participants who had recently been hospitalised or had caregiving experience, and (c) analysis of Voice of Customer data from a large general hospital [11, 12]. This qualitative phase identified important aspects of patient experience specific to the South Korean healthcare context, such as the ease of raising complaints when needed, opportunities to communicate with doctors, and information about doctors' hospital rounds [12]. Through this process, an initial pool of 31 items was generated.

#### 2.1.2 Phase 2: Item Review (2014)

The initial 31 items underwent expert review using the Delphi method. A panel of 21 experts (including doctors, nurses, hospital communication specialists, public hospital directors, and professors) evaluated each item for clarity, relevance, and appropriateness. Based on their feedback, we reviewed and revised the items to ensure content validity, which led to a selected set of 24 items for pilot testing [11].

#### 2.1.3 Phase 3: Initial Validity and Reliability Testing (2015)

A pilot study was conducted in three general hospitals in Seoul in February 2015. Trained interviewers administered the survey either face‐to‐face or via telephone, depending on each hospital's policy. Factor analysis was conducted to assess construct validity, and reliability was evaluated using internal consistency measures. Factor analysis identified five constructs from 21 items. These five empirically derived constructs served as the conceptual foundation for five substantive domains. In addition, two global items were included as a separate domain to assess overall patient experience. Thus, the initial complete version of the PXA questionnaire consisted of 23 items across six domains. The analysis demonstrated satisfactory levels of validity and reliability. A detailed description of the tool development and validation process is available in the Health Insurance Review and Assessment Service (HIRA) policy report [11].

### 2.2 Practical Refinement During Nationwide Survey Implementation (2016–2022)

During preparation for the first nationwide implementation in 2017, practical refinements were made based on stakeholder feedback regarding survey feasibility. Three items with overlapping content were consolidated or removed, resulting in a reduction from 23 to 21 items. Following the first implementation in 2017, the response scale of Q13 (Information about post‐discharge care) was revised from a four‐point Likert scale to a binary (yes/no) format based on survey results and stakeholder discussions.

The revised PXA questionnaire consists of 21 items across six domains: Communication with nurses (Q1–Q4), Communication with doctors (Q5–Q8), Medication and treatment (Q9–Q13), Hospital environment (Q14–Q15), Patient rights (Q16–Q19), and Overall rating (Q20–Q21) [18]. The first five domains (19 items, Q1–Q19) maintain the conceptual structure based on the five constructs identified in the initial factor analysis. The Overall rating domain contains two global items (Q20–Q21) that serve as summary measures of overall patient experience. Most items use a 4‐point Likert scale, with Q13 using a binary scale and global items (Q20–Q21) using a 0–10 scale. Three items (Q11, Q17, Q19) include a ‘Not Applicable’ response option. These modifications, along with evolving sociocultural and healthcare environments relevant to patient experience during this implementation period, prompted the need to reassess the tool's conceptual and structural validity.

### 2.3 Reassessment of Psychometric Properties (2022)

This section describes the methods used in the current study to reassess the PXA's psychometric properties, which is the primary focus of this paper.

#### 2.3.1 Data Collection

We conducted a cross‐sectional survey to reassess the psychometric properties of the current version of the PXA questionnaire. Based on established guidelines for factor analysis, a minimum sample size of approximately 200–300 was required [20, 21, 22, 23, 24, 25]. Given the previous survey's response rate of approximately 10%, we set a recruitment target of 4800 inpatients, which would substantially exceed the recommended minimum, even accounting for non‐response.

To ensure feasibility while capturing diversity in hospital characteristics, participating hospitals were selected based on the following criteria: (1) established administrative capacity to obtain patient consent for research participation during admission, (2) sufficient patient volume to achieve recruitment targets within the study timeline, and (3) inclusion of at least one hospital outside the Seoul metropolitan area. Based on these criteria, four general hospitals were selected with varying capacities: two with more than 1,000 beds (Hospitals A and B), one with 500–999 beds (Hospital C), and one with fewer than 500 beds (Hospital D).

Following the eligibility criteria defined in the PXA, the study included adult patients (aged ≥ 19 years) who had been hospitalised for at least 1 day and completed the survey within 2 to 56 days after discharge, having provided consent for data use. Patients from the obstetrics and gynaecology, psychiatry, and paediatrics departments were excluded.

Data collection was performed over a 4‐week period from October to November 2022. The survey comprised 19 patient experience items (Q1–Q19), two global items (Q20–Q21), and three additional patient characteristics not captured in hospital admission records: admission type, self‐rated health, and education level. A total of 1520 patients completed the survey.

#### 2.3.2 Analysis Strategy

To explore the underlying dimensions and structure of the PXA, we first conducted an exploratory factor analysis (EFA), followed by a confirmatory factor analysis (CFA) to evaluate the stability and model fit of the factor structure identified in the EFA. A split‐sample approach was employed to enhance validation through cross‐validation, minimising the influence of sample‐specific results [20, 21, 22]. The total sample was randomly divided into two equal subsamples to use for EFA and CFA, respectively. To confirm the equivalence of the two subsamples, we performed a chi‐square test on socio‐demographic variables, including hospital of origin, survey mode, gender, age group, admission type, self‐rated health, and education level. Additionally, we assessed inter‐factor and item‐factor correlations to further investigate the relationships between individual items and their respective factors. Corrected item‐total correlations and Cronbach's alpha were used for reliability estimation [22].

The analysis encompassed items Q1–Q19, excluding Q13 due to its dichotomous response format. Preliminary analysis supported this exclusion, as Q13 demonstrated weak correlations with other items (*r* = 0.15–0.35) and low communality (*h²* = 0.15). The ‘Not Applicable’ option was provided for items where certain services may not be relevant to all patients (e.g., ‘Q11. Efforts to control pain’ were not applicable to patients who experienced no pain during hospitalisation). These responses represent legitimate skip patterns based on individual care experiences. Given the sufficient sample size for each subsample (> 300) [20, 21, 22, 23, 24, 25], we applied listwise deletion for missing (*n* = 16) and ‘Not Applicable’ responses (*n* = 875), yielding analytical samples of 315 and 314 for EFA and CFA, respectively.

#### 2.3.3 Validity

##### 2.3.3.1 Exploratory Factor Analysis (EFA)

We assessed data suitability using the Kaiser‐Meyer‐Olkin (KMO) measure and Bartlett's test of sphericity. Data were considered appropriate for factor analysis when the KMO value exceeded 0.70, and Bartlett's test yielded a *p*‐value less than 0.05 [20, 21, 24].

Given that our data did not assume a normal distribution, we employed Principal Axis Factoring as the extraction method, which provides optimal results for non‐normally distributed data [23, 24]. We applied oblique oblimin rotation, assuming correlations between factors. The optimal number of factors was determined based on parallel analysis results [22, 26]. We interpreted the factor structure using a factor loading cut‐off of 0.40 [20].

##### 2.3.3.2 Confirmatory Factor Analysis (CFA)

To perform factor analysis with ordinal data, we used diagonally weighted least squares estimation [27]. Standardised factor loadings above 0.70 were considered ideal for establishing convergent validity [20]. Discriminant validity was deemed adequate when the average variance extracted for each construct was greater than the squared correlations with any other construct [20]. We report the comparative fit index (CFI), Tucker‐Lewis index (TLI), root mean square error of approximation (RMSEA), and standardised root mean square residual (SRMR) as model fit indices. Models were considered to have an adequate fit if CFI and TLI values exceeded 0.95, RMSEA was below 0.06, and SRMR was less than 0.08 [28].

##### 2.3.3.3 Inter‐Factor and Item‐Factor Correlation

These correlations were computed based on the final factor structure in our study, providing insight into the scale's structure and item relationships [22]. Inter‐factor and item‐factor correlations were assessed using Spearman's correlation coefficients. We examined whether items correlated more strongly with their own factor than with other factors.

#### 2.3.4 Reliability

Corrected item‐total correlations were calculated to assess internal consistency. Each item is correlated with the total score excluding that item, providing evidence of the scale's internal consistency. Coefficients of 0.30 or higher were considered acceptable, with values above 0.40 indicating a good association between the item and the overall scale [22]. Cronbach's alpha was also used as a measure of internal consistency. Values exceeding 0.70 were considered acceptable indicators of reliability [20, 28].

## 3 Results

### 3.1 Participants

Among the respondents who completed the survey questionnaire (*n* = 1520), data from 629 individuals (41.3%) were included in the analysis after excluding cases with any missing (*n* = 16) or ‘Not Applicable’ responses (*n* = 875) to ensure the robustness of factor analysis. The sample was divided into two subsamples (*n* = 315 for the EFA and *n* = 314 for the CFA). The chi‐squared test revealed no significant distribution difference between the two subsamples. The characteristics of the respondents are presented in Table 1.

**TABLE 1:** Summary statistics.

Characteristics	Total		EFA		CFA		*p* †
	*N*	(%)	*N*	(%)	*N*	(%)	
	***629***	**(100.0)**	***315***	**(100.0)**	***314***	**(100.0)**	
**Hospital of origin**							0.94
A	216	(34.3)	111	(35.2)	105	(33.4)	
B	211	(33.5)	106	(33.7)	105	(33.4)	
C	130	(20.7)	64	(20.3)	66	(21.0)	
D	72	(11.4)	34	(10.8)	38	(12.1)	
**Survey mode**							
Mobile	456	(72.5)	223	(70.8)	233	(74.2)	0.39
Telephone	173	(27.5)	92	(29.2)	81	(25.8)	
**Gender**							
Male	306	(48.6)	148	(47.0)	158	(50.3)	0.45
Female	323	(51.4)	167	(53.0)	156	(49.7)	
**Age group**							
20–29	29	(4.6)	17	(5.4)	12	(3.8)	0.83
30–39	55	(8.7)	25	(7.9)	30	(9.6)	
40–49	134	(21.3)	66	(21.0)	68	(21.7)	
50–59	122	(19.4)	64	(20.3)	58	(18.5)	
60–69	176	(28.0)	88	(27.9)	88	(28.0)	
70–79	94	(14.9)	48	(15.2)	46	(14.6)	
80+	19	(3.1)	7	(2.2)	12	(3.8)	
**Admission type**							
Via the other means	468	(74.4)	235	(74.6)	233	(74.2)	0.60
Via the Emergency department	160	(25.4)	79	(25.1)	81	(25.8)	
‘Do not know’ or refusal to respond	1	(0.2)	1	(0.3)	0	(0.0)	
**Self‐rated health**							
Very poor	55	(8.7)	32	(10.2)	23	(7.3)	0.45
Poor	178	(28.3)	86	(27.3)	92	(29.3)	
Moderate	250	(39.7)	131	(41.6)	119	(37.9)	
Good	125	(19.9)	58	(18.4)	67	(21.3)	
Very good	20	(3.2)	8	(2.5)	12	(3.8)	
‘Do not know’ or refusal to respond	1	(0.2)	0	(0.0)	1	(0.4)	
**Education level**							
Middle school or less	90	(14.3)	50	(15.9)	40	(12.7)	0.46
High school	227	(36.1)	114	(36.2)	113	(36.0)	
College (still attending)	13	(2.1)	6	(1.9)	7	(2.2)	
College	242	(38.5)	120	(38.1)	122	(38.9)	
Graduate school	55	(8.7)	23	(7.3)	32	(10.2)	
‘Do not know’ or refusal to respond	2	(0.3)	2	(0.6)	0	(0.0)	

^†^ These results indicated *p*‐value from chi‐square tests comparing demographics between EFA and CFA samples. Statistical significance was defined as *p* < 0.05

### 3.2 Psychometric Properties

#### 3.2.1 Validity

##### 3.2.1.1 Exploratory Factor Analysis (EFA)

The data were suitable for factor analysis (KMO = 0.95, Bartlett's test *p* < 0.001). Parallel analysis suggested a four‐factor solution. The four factors explained 67% of the total variance, with factor loadings ranging from 0.41 to 0.95. This empirically derived factor structure was also conceptually sound. The factors were labelled as follows: Information, dignity, and autonomy (Factor 1), Communication with nurses (Factor 2), Communication with doctors (Factor 3), and Hospital environment (Factor 4) (Table 2).

**TABLE 2:** Factor loadings and communalities (*h* ^2^) from exploratory factor analysis (*N* = 315).

Items	Factor loadings†				*h* ^2^
	1	2	3	4	
Q1. Courtesy and respect of nurses	0.02	**0.70**	–0.05	0.13	0.59
Q2. Attentive listening of nurses	0.00	**0.88**	0.09	–0.04	0.84
Q3. Understandable explanations of nurses	0.13	**0.68**	0.06	0.03	0.70
Q4. Commitment to handling requests of nurses	0.09	**0.69**	0.09	0.05	0.71
Q5. Courtesy and respect of doctors	0.10	0.05	**0.78**	0.05	0.81
Q6. Attentive listening of doctors	–0.02	0.08	**0.91**	0.02	0.90
Q7. Opportunities to communicate with doctors	**0.55**	–0.16	0.36	0.09	0.64
Q8. Information about doctor's hospital rounds	**0.47**	0.09	0.18	0.06	0.51
Q9. Explanation about medication/test/treatment	**0.74**	0.15	–0.03	0.01	0.71
Q10. Explanation about possible side effects	**0.86**	0.01	–0.01	–0.04	0.69
Q11. Efforts to control pain	**0.61**	0.12	0.13	0.02	0.66
Q12. Emotional support	**0.55**	0.10	0.19	0.08	0.67
Q14. Overall cleanliness	0.01	–0.07	0.02	**0.95**	0.87
Q15. Overall safety	–0.01	0.23	0.00	**0.70**	0.71
Q16. Fair treatment	**0.41**	0.32	–0.10	0.20	0.57
Q17. Ease of raising complaints when needed	**0.43**	0.14	0.06	0.20	0.52
Q18. Opportunities for shared decision making	**0.72**	–0.07	0.08	0.04	0.57
Q19. Consideration to prevent shame	**0.65**	0.07	–0.14	0.05	0.42
**Proportion Var**	0.26	0.18	0.12	0.11	—
**Cumulative Var**	0.26	0.44	0.56	0.67	—

*Note:* The extraction method was Principal Axes Factor (PAF). The values of factor loadings are derived from the pattern matrix, with loadings equal to or greater than 0.4 are highlighted in bold.

^†^ Each factor was labelled as follows: Information, dignity, and autonomy (Factor 1), Communication with nurses (Factor 2), Communication with doctors (Factor 3), Hospital environment (Factor 4).

##### 3.2.1.2 Confirmatory Factor Analysis (CFA)

**FIGURE 1:**
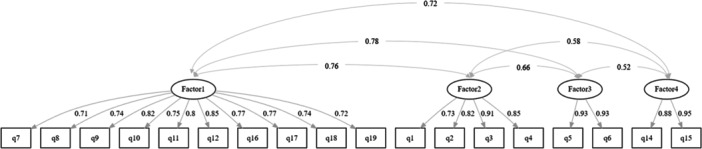
Diagram of CFA with standardised parameter estimates. *Note:* In this path diagram, ellipses represent latent variables (factors), rectangles indicate observed variables (items), and arrows show the relationships between variables. Single‐headed arrows from latent factors to observed variables represent standardised factor loadings, while double‐headed arrows between latent factors indicate inter‐factor correlations. All standardised factor loadings exceeded 0.70 (the ideal threshold for convergent validity).

CFA was conducted based on the four‐factor structure generated by EFA. Figure 1 visualises the CFA results, demonstrating high convergent validity with standardised loading values exceeding 0.70 for all items (range: 0.71–0.95). These results indicate that the PXA instrument demonstrates appropriate measurement and satisfactory convergent validity. The discriminant validity indicated that most factors are fairly exhibited, as their AVE values exceed squared correlations. Although a minor discriminant validity concern arose in the Factor 1–Factor 3 relationship (AVE = 0.59 < *R* ^2^ = 0.60), an alternative three‐factor model combining Factor 1 and Factor 3 did not yield a valid factor structure. Moreover, given the theoretical distinctiveness of the constructs measured by Factor 1 and Factor 3, maintaining the current four‐factor structure offers the most theoretically sound and empirically adequate solution. The final model demonstrated satisfactory fit indices (*χ*² = 230.128, df = 129, *p* < 0.001, Robust CFI and TLI = 1.000 and 1.004, Robust RMSEA = 0.036, SRMR = 0.046).

##### 3.2.1.3 Inter‐Factor and Item‐Factor Correlation

Analysis of the correlation matrix, as presented in Table 3, revealed that each item demonstrated a stronger correlation with its associated factor compared to other factors. These findings align with the theoretical expectation that items should correlate more strongly with their own factors.

**TABLE 3:** Correlations between factors and items, and internal consistency (*n* = 629).

Factors and items	Inter‐factor and item‐factor correlations†				Corrected item‐total correlations‡	Cronbach's alpha
	1	2	3	4		
**Information, dignity, and autonomy**	**1.00**					
Q7. Opportunities to communicate with doctors	0.80	0.52	0.65	0.49	0.71	0.93
Q8. Information about doctor's hospital rounds	0.79	0.57	0.60	0.52	0.73	
Q9. Explanation about medication/test/treatment	0.83	0.67	0.63	0.61	0.80	
Q10. Explanation about possible side effects	0.81	0.62	0.61	0.56	0.75	
Q11. Efforts to control pain	0.80	0.69	0.64	0.60	0.79	
Q12. Emotional support	0.85	0.66	0.66	0.59	0.81	
Q16. Fair treatment	0.76	0.65	0.56	0.64	0.75	
Q17. Ease of raising complaints when needed	0.80	0.60	0.58	0.59	0.75	
Q18. Opportunities for shared decision making	0.80	0.56	0.60	0.54	0.74	
Q19. Consideration to prevent shame	0.76	0.61	0.56	0.55	0.72	
**Communication with nurses**	**0.73**	**1.00**				
Q1. Courtesy and respect of nurses	0.57	0.85	0.51	0.52	0.65	0.90
Q2. Attentive listening of nurses	0.65	0.88	0.60	0.50	0.72	
Q3. Understandable explanations of nurses	0.68	0.89	0.62	0.58	0.75	
Q4. Commitment to handling requests of nurses	0.66	0.87	0.58	0.55	0.72	
**Communication with doctors**	**0.75**	**0.66**	**1.00**			
Q5. Courtesy and respect of doctors	0.72	0.64	0.96	0.54	0.76	0.93
Q6. Attentive listening of doctors	0.72	0.64	0.97	0.50	0.75	
**Hospital environment**	**0.67**	**0.61**	**0.54**	**1.00**		
Q14. Overall cleanliness	0.63	0.56	0.51	0.96	0.67	0.89
Q15. Overall safety	0.65	0.61	0.53	0.95	0.70	

*Note:* All correlations are Spearman's rank correlations and were statistically significant (*p* < 0.001).

^†^ Bold values represent inter‐factor correlations, while others represent item‐factor correlations. These correlations are not corrected for item overlap with the factor total.

^‡^ Corrected item‐total correlations indicate the relationship between each item and the total score with that item excluded from the total. This correction prevents artificial inflation of the correlation due to item overlap.

#### 3.2.2 Reliability

The reliability analysis results are presented in Table 3. All items showed good corrected item‐total correlations (*r* > 0.40), with ‘Emotional support’ (Q12) having the strongest correlation. The instrument demonstrated high internal consistency, with a Cronbach's alpha of 0.95 for the entire scale and ranging from 0.89 to 0.93 for individual subscales.

## 4 Discussion

This study analysed the psychometric properties of the Patient Experience Assessment (PXA) questionnaire, a national survey instrument developed for use in South Korea. EFA revealed a four‐factor structure: (1) Information, dignity, and autonomy; (2) Communication with nurses; (3) Communication with doctors; and (4) Hospital environment. CFA supported this structure, demonstrating high convergent validity, with standardised loading values exceeding 0.70 for all items. The model also satisfied all goodness‐of‐fit indices (CFI and TLI > 0.950, RMSEA = 0.036, SRMR = 0.046). The PXA questionnaire also showed excellent reliability, with a Cronbach's alpha of 0.95 for the entire instrument and 0.89 to 0.93 across subscales, indicating high internal consistency. These findings confirm that the PXA questionnaire is a reliable and valid instrument for assessing patient experiences in the South Korean healthcare context.

The current four‐factor structure demonstrates substantial continuity with the original five‐factor structure identified during initial validation in 2015 [18]. The original validation, conducted with 21 items from three Seoul hospitals, identified five empirical factors that served as the conceptual foundation for five substantive domains: Communication with nurses, Communication with doctors, Medication and treatment, Hospital environment, and Patient rights. In the current reassessment, three factors remained conceptually identical: Communication with nurses (Factor 2), Communication with doctors (Factor 3), and Hospital environment (Factor 4). The primary difference lies in the convergence of the original ‘Medication and treatment’ and ‘Patient rights’ domains into a single ‘Information, dignity, and autonomy’ dimension (Factor 1). This convergence likely reflects two influences: (1) the reduction from 21 to 18 analysed items may have decreased statistical power to distinguish closely related dimensions, and (2) patients may increasingly perceive information provision, treatment involvement, and respectful care as an integrated dimension rather than separate aspects. Despite this convergence, the substantial overlap—three of four current factors directly corresponding to original factors—demonstrates structural stability over 7 years of implementation, supporting the PXA's continued validity for measuring patient experience in hospital inpatient care in South Korea.

### 4.1 Comparison With Previous Studies

The PXA questionnaire effectively integrates widely recognised conceptual themes and key elements of patient‐centeredness while addressing the unique needs of Korean patients.

Validated patient experience instruments across multiple healthcare systems consistently measure the following core domains: communication with doctors and nurses, information and education, physical comfort and pain management, hospital environment, discharge planning, staff responsiveness, and overall rating [5, 6, 7, 8, 9, 10, 14, 15, 29, 30, 31, 32]. These domains reflect universal principles of patient‐centred care. The PXA encompasses these widely recognised themes and established domains. Communication with nurses (Factor 2) and Communication with doctors (Factor 3) correspond to communication domains commonly distinguished in international instruments. Hospital environment (Factor 4) aligns with the environmental quality domain. Information, dignity, and autonomy (Factor 1) encompasses elements of information and education, physical comfort and pain management, discharge planning, and staff responsiveness, integrating these with broader themes of respect for patient preferences and values.

Additionally, the PXA also addresses unique characteristics of the South Korean healthcare system. In the South Korean context, where persistent shortages of medical personnel are well documented, patients often report limited opportunities for interaction with providers [12, 18, 19]. Moreover, hierarchical patient‐provider relationships characteristic of Korean healthcare culture create barriers to raising complaints [12]. To capture these experiences, the PXA questionnaire includes items, such as Information about doctor's hospital rounds (Q8) and Ease of raising complaints when needed (Q17), that have received relatively little attention in patient experience measurement instruments used in other countries. Indeed, according to the results of the fourth national PXA in 2023, these items consistently received lower scores compared to other items. This persistent gap indicates unmet patient expectations and highlights opportunities for improvement.

These findings align with our expectations and emphasise the importance of considering patient needs and expectations in both developing patient experience measurements and implementing quality improvement initiatives [3]. Patient experiences, needs, and expectations are inherently shaped by sociocultural context and healthcare system structures [16, 17]. Consequently, measurement tools developed without local patient input risk overlooking the care aspects that patients prioritise most. Given that patient‐centred care aims to meet individual patients' needs and preferences [2, 16, 31], our results provide evidence that patient engagement in developing patient experience measurements and identifying priorities for assessment is not merely methodologically sound but operationally essential [3]. The PXA demonstrates how assessment tools can effectively integrate well‐established dimensions of patient‐centredness with culture‐specific healthcare needs, offering valuable insights for other healthcare systems seeking to develop contextually relevant assessment instruments.

### 4.2 Policy Implications

Our factor analysis revealed a four‐factor structure that differs from the 5 domains (excluding the Overall rating domain) currently used in PXA scoring. This misalignment between the statistically derived factor structure and the existing scoring domains suggests that domain‐specific composite scores may not accurately reflect the intended constructs. Since 2017, the HIRA has used the PXA questionnaire to evaluate and publicly report national‐level patient experience results, including domain‐level composite scores. While this composite scoring approach offers practical benefits by condensing data presentation, our results suggest caution is warranted in the current domain‐specific scoring approach. To address this concern, one possible solution is to provide individual item scores alongside composite scores to enhance the transparency of specific aspects of patient experience. Additionally, we recommend developing detailed interpretation guidelines with an improved user interface that acknowledges the complex factor structure identified in this study.

These strategies could contribute to a more accurate and meaningful interpretation of PXA results for stakeholders and policymakers. The observed structural discrepancies likely stem from item reduction during implementation and reflect an inherent tension in patient experience measurement: statistically‐derived structures identify empirical patterns among variables, while conceptual domains represent theoretical frameworks for interpretation and practical application. Statistical fit alone does not guarantee conceptual relevance—empirical patterns must be interpreted through conceptual lenses to ensure meaningful use [20, 21, 22]. This tension between statistical findings and conceptual organisation represents a broader challenge where both analytical rigour and interpretive validity are essential. Future surveys should incorporate both frameworks: empirically‐derived structures for statistical analysis and conceptual domains for reporting and interpretation, with explicit documentation of their relationship.

### 4.3 Limitations and Implications for Future Research

This study provides a comprehensive analysis of the psychometric properties of the PXA questionnaire in South Korea. While numerous studies have examined the validity of patient experience instruments in various countries [7, 8, 9, 10, 14, 15, 29, 30, 31, 32], such research has been lacking in the South Korean context. By addressing this knowledge gap, this study contributes to the global discourse on the development and validation of patient experience measurement tools.

However, several limitations should be noted, each of which points to directions for future research. First, two of the factors (Factors 3 and 4) in our final model consist of only two sub‐items each. While this does not meet the general criteria for factor analysis [22], it is statistically and conceptually justified in our case. Statistically, two‐item factors are considered acceptable when items demonstrate high inter‐item correlations (*r* > 0.70), while maintaining distinctly lower correlations with other variables [24, 25]; our preliminary correlation analysis confirmed that inter‐item correlations exceeded 0.70, while remaining distinctly higher than correlations with other items. Our reliability analysis (Table 3) further confirms these criteria: both factors demonstrated high internal consistency (Cronbach's *α* = 0.93 for Factor 3, 0.89 for Factor 4) and high item‐factor correlations (> 0.95). Moreover, the substantial gaps between within‐factor correlations and cross‐factor correlations (differences of 0.24–0.30) establish strong discriminant validity. Conceptually, each factor represents a distinct and interpretable dimension of patient experience. Nevertheless, future research may consider developing additional items to strengthen these factors [20]. Second, item Q13 was excluded from the analysis due to its dichotomous response format and weak psychometric properties (low communality and correlations with other items). However, Q13 was retained in the operational PXA questionnaire because it captures an important aspect of patient experience related to transitions of care, which remains conceptually valuable despite its weak statistical integration with other items. Future research should consider alternative analytical approaches, such as item response theory or separate validation methods for dichotomous items, to better integrate this item into psychometric validation while preserving its conceptual contribution. Third, there is potential for selection bias stemming from our handling of missing data. We excluded cases with ‘Not Applicable’ responses, which indicated that certain services or care experiences were not part of the respondent's care pathway. This means our analysis included only patients who experienced all services covered by the PXA questionnaire items. If the responses are systematically associated with specific respondent characteristics or treatment pathways, the resulting factor structure may not generalise to the broader target population, particularly patients with limited service exposure. Future studies could employ analytical approaches that accommodate structural non‐response, such as conducting separate confirmatory factor analyses within patient subgroups defined by service exposure patterns, using only relevant items for each subgroup. Fourth, although our sample included four general hospitals with varying bed capacities and geographic locations, it did not encompass smaller healthcare facilities with fewer than 300 beds. As such, caution should be exercised when generalising these findings. Fifth, while this study demonstrated the robustness of the PXA's structural validity and internal consistency, additional measurement properties remain to be evaluated to establish the instrument as a sound tool for generating valid and comparable results across hospital levels [28]. These include measurement invariance across hospital levels and demographic subgroups, test‐retest reliability, and responsiveness to changes over time. Future research should examine these properties to ensure the validity of cross‐group comparisons and support the PXA's application in diverse contexts.

## 5 Conclusion

This study outlined the development process and evaluated the psychometric properties of the PXA questionnaire, a tool that integrates globally established dimensions of patient‐centredness with context‐specific aspects of the South Korean healthcare system. The findings confirm a stable four‐factor structure with satisfactory validity and reliability, demonstrating that the PXA questionnaire remains psychometrically robust. These results highlight the continued utility of the PXA questionnaire as a reliable tool for assessing patient experience. Furthermore, the development and validation process offers valuable guidance for other healthcare systems seeking to design culturally relevant patient experience measures.

## Author Contributions

**Yeongchae Song:** conceptualisation (supporting), investigation (supporting), data curation (equal), methodology (equal), formal analysis (lead), project administration (supporting), writing – original draft (lead), writing – review and editing (supporting). **Jung‐Eun Kim:** conceptualisation (supporting), methodology (equal), writing – original draft (supporting), writing – review and editing (supporting). **Bon Mi Koo:** investigation (supporting), data curation (equal), project administration (supporting), writing – review and editing (supporting). **Young‐Geun Choi:** investigation (supporting), methodology (supporting), validation (lead), writing – review and editing (supporting). **Un‐Na Kim:** investigation (supporting), writing – review and editing (supporting). **Jin Yong Lee:** investigation (supporting), writing – review and editing (supporting). **Min‐Woo Jo:** investigation (supporting), writing – review and editing (supporting). **Minsu Ock:** investigation (supporting), writing – review and editing (supporting). **Young Kyung Do:** supervision (lead), funding acquisition (lead), conceptualisation (lead), investigation (lead), project administration (lead), writing – review and editing (lead).

## Ethics Statement

The study was approved by the Institutional Review Boards of Seoul National University Hospital (IRB No. H‐2207‐134‐1342), Asan Medical Centre (IRB No. 2022‐1284), Ulsan University Hospital (IRB No. UUH2022‐07‐043), and Dongguk University Gyeongju Hospital (IRB No. 110757‐202207‐h‐03‐04). This study only included patients who had provided written informed consent for the use of their personal information through the respective hospital's consent form at the time of admission, ensuring voluntary participation in accordance with ethical research guidelines.

## Conflicts of Interest

The authors declare no conflicts of interest.

## Supporting information

Supplementary_Material_for_Review.

## Data Availability

The data for this study were collected by the research team as part of a contract research project commissioned by the Health Insurance Review and Assessment Service. Data availability requests should be made to the Health Insurance Review and Assessment Service.
