# Supplementary material for: Developing the Patient Experience Assessment Questionnaire for Hospital Inpatient Care in South Korea
Source: Health Expect. 2026 Feb 3;29(1):e70560. doi: 10.1111/hex.70560 (PMC12868926; doi:10.1111/hex.70560)

**Appendix.** Timeline of PXA questionnaire development, national survey implementation, and psychometric reassessment


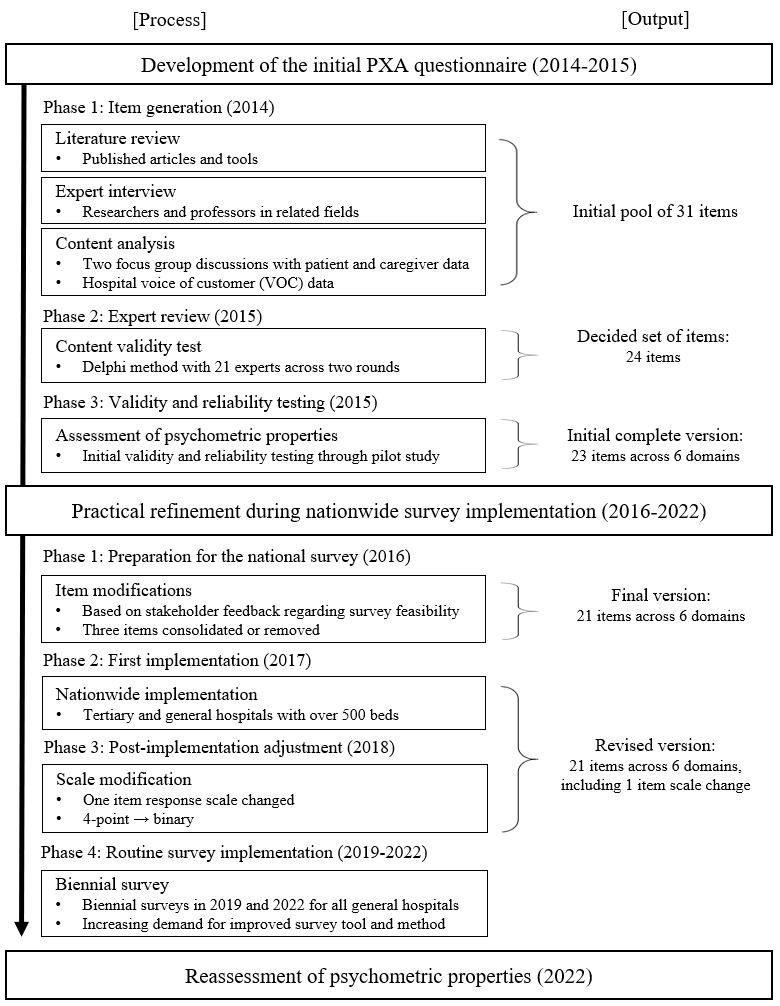

Supplement: Supplementary file 1 — Supplementary_Material_for_Review. [file HEX-29-e70560-s001.docx]
